# Literature Mining Solutions for Life Science Research

**DOI:** 10.1155/2013/320436

**Published:** 2013-01-08

**Authors:** Jörg Hakenberg, Goran Nenadic, Dietrich Rebholz-Schuhmann, Jin-Dong Kim

**Affiliations:** ^1^Disease Translational Informatics, F. Hoffmann-La Roche Inc., Nutley, NJ 07110, USA; ^2^School of Computer Science and Manchester Institute of Biotechnology, University of Manchester, Manchester M13 9PL, UK; ^3^European Bioinformatics Institute, Wellcome Trust Genome Campus, Hinxton, Cambridge CB10 1SD, UK; ^4^Institut für Computerlinguistik, Universität Zürich, 8050 Zürich, Switzerland; ^5^Database Center for Life Science (DBCLS), Research Organization of Information and Systems (ROIS), Tokyo 113-0032, Japan

Research and development in the area of biomedical literature analysis aims at providing life science researchers with effective means to access and exploit knowledge contained in scientific publications. Virtually all journal publications and many conference proceedings are nowadays readily available in an electronic form—for instance, as abstracts through the MEDLINE citation index or as full-text articles through PubMed Central. Nevertheless, keeping up to date with and searching for recent findings in a research domain remains a tedious task hampered by inefficient and ineffective means for access and exploitation. Biomedical text analysis aims to improve access to unstructured knowledge by alleviating searches, providing auto generated summaries of documents and topics, linking and integrating publications with structured resources, visualizing content for better understanding, and guiding researchers to novel hypotheses and into knowledge discovery.

Focused research over recent years has improved fundamental solutions for biomedical text mining, such as document retrieval, named entity recognition, normalization and grounding, and extraction of relationships, with levels of accuracy that reach human annotators when considering inter annotator agreement. Consequently, more and more integrative analysis tools were put forward by the text mining community targeting a broad audience of end users: generic and task-specific search engines for life science researchers, interfaces for networks synthesis based on textual evidences, or more specialized tools searching for transcription factors, or primer sequences.

This special issue of Advances in Bioinformatics presents overviews and examples of end-user-oriented biomedical text mining tools for bioinformaticians, molecular biologists, biochemists, clinicians, pharmacologists, and other researchers in life sciences. 

We start with A. Manconi et al. survey on “*Literature retrieval and mining in bioinformatics: state of the art and challenges.*” The authors introduce the major concepts that life science researchers should be familiar with getting the best out of existing text mining solutions, and survey key tools and research. In a dedicated second part of their survey, the authors address the major challenges both life science researchers and solution developers are facing at this point. The reader will find plenty of references to existing search tools, resources, and research papers.

A. E. Thessen et al. focus on a particular domain, presenting an overview of “*Applications of natural language processing in biodiversity science.*” The authors review the application of natural processing and machine learning for biological information extraction regarding cellular processes, taxonomic names, and morphological characters. You will find detailed examples, a summary of all steps involved in information extraction, and lots of references to existing tools and resources.

S. T. Ahmed et al. introduce their semantic faceted search engine, BioEve, in “*A novel framework to facilitate interactive literature search.*” They couple an automated extraction system with a cognitive search and navigation service, to alleviate the process of searching and browsing huge amounts of literature such as provided/delivered by MEDLINE. BioEve enables interactive query refinement and suggests concepts and entities (like genes, drugs, and diseases) to quickly filter and modify search directions, thereby achieving semantic enrichment that improves insight gains while searching literature.

S. V. Landeghem et al. present their EVEX resource in “*Exploring biomolecular literature with EVEX: connecting genes through events, homology, and indirect associations.*” The authors extracted more than 20 million biomolecular events involving genes and proteins, such as phosphorylation and gene regulation, from MEDLINE. The online tool generates a summary on the searched gene denoting all regulated genes, binding partners, subcellular locations, and other related data linked to the searched gene.

We conclude this special issue with the paper by A. Divoli et al., discussing whether “*Do Peers see more in a paper than its authors.*” In a meta-analysis using automatic text analysis, they address questions such as how informative an abstract is compared to the full text; and how peers and authors might view the major contributions of a paper differently. Their analyses are comparing the information content of an abstract, as written by the paper's authors, to sentences that mention the paper as a reference, written by peers. Using this strategy, A. Divoli et al. found, for example, that citing sentences contain 20% additional concepts (likely important contributions) that were not mentioned in the abstract of the paper referred to, but maybe should have been to help attract even more peers.

